# Age-related prevalence and characteristics of *Aggregatibacter actinomycetemcomitans* in periodontitis patients living in Sweden

**DOI:** 10.1080/20002297.2017.1334504

**Published:** 2017-06-20

**Authors:** Rolf Claesson, Carola Höglund-Åberg, Dorte Haubek, Anders Johansson

**Affiliations:** ^a^ Division of Oral Microbiology, Department of Odontology, Umeå University, Umeå, Sweden; ^b^ Division of Molecular Periodontology, Department of Odontology, Umeå University, Umeå, Sweden; ^c^ Section for Pediatric Dentistry, Department of Dentistry, Health, Aarhus University Aarhus, Denmark

**Keywords:** *Aggregatibacter actinomycetemcomitans*, leukotoxin, JP2, serotypes, AP-PCR genotypes, microbiological diagnostics

## Abstract

**Background**: The presence of *Aggregatibacter actinomycetemcomitans* in patients with periodontitis has been extensively studied for decades.

**Objective**: To study the prevalence of *A. actinomycetemcomitans* in younger and older periodontitis patients and to genetically characterize isolates of this bacterium.

**Design**: Data from microbiological analyses of 3459 subgingival plaque samples collected from 1445 patients, 337 ‘younger’ patients (≤35 yrs) and 1108 ‘older’ patients (>35 yrs) during 15 years (2000–2014), has been summerized. Isolates of *A. actinomycetemcomitans* were serotyped, leukotoxin promoter typed (JP2 and non JP2) and arbitrarily primed PCR (AP-PCR) genotyped. The origin of the JP2 genotype detected in the study population was determined.

**Results**: The prevalence of *A. actinomycetemcomitans* was higher among younger than older patients and samples from the younger patients contained higher proportions of the bacterium. Serotype b was more prevalent among younger patients and the majorty of these isolates was from the same AP-PCR genotype. The JP2 genotype was detected in 1.2% of the patients, and the majority of these carriers were of non-African origin.

**Conslusions**: For presence and charcteristics of *A. actinomycetemcomitans* in clinical samples the age of the carriers were a discriminating factor. Additional, apparently non-African carriers of the JP2 genotype of *A. actinomycetemcomitans* were identified.

## Introduction

Periodontitis is an infectious disease with a concomitant host response associated with the destruction of the periodontium [1]. In contrast to many other infections, periodontitis is rarely associated with one or a few bacterial species; it is rather a polyinfection, with a collection of different oral microorganisms involved [2–5].

The bacterium *Aggregatibacter actinomycetemcomitans* is strongly associated with aggressive forms of the disease [6]. Based on longitudinal studies, the presence of this bacterium is considered a marker for progression of periodontal attachment loss (AL), that is, degradation of periodontal tissues around the teeth [7].

Seven serotypes (a–g) of *A. actinomycetemcomitans* have been described [8]. The prevalence of these serotypes is associated with a number of factors, such as geographic localization, ethnicity, and periodontal status of the carriers [8,9]. Among the serotypes, a, b, and c are the most common. Serotype b is more pathogenic due to its higher capacity to produce leukotoxin, the main virulence factor of *A. actinomycetemcomitans* [10–12]. The leukotoxin kills neutrophils and macrophages in cellular processes associated with the activation and release of proteases and interleukin-1β (IL-1β), respectively [13]. These properties of the leukotoxin are believed to attenuate the host response and predispose to periodontal breakdown [13].

Very high levels of leukotoxin production have been detected in serotype b isolates that lack a 530 base-pair (bp) DNA fragment within the leukotoxin promoter region [14,15]. Isolates with this characteristic are described as the JP2 genotype of *A. actinomycetemcomitans* [14], whereas the non-JP2 genotype has a full-length leukotoxin promoter region. Carriers of the JP2 genotype are at increased risk of developing periodontal disease compared to carriers of the non-JP2 genotype [16,17].

The JP2 genotype of *A. actinomycetemcomitans* emerged on the African continent >2,000 years ago, and since then, it has spread worldwide through human migration of African populations [18]. Although the JP2 genotype today can be detected in areas far from Africa, the carriers have been almost exclusively of African descent. However, a few exceptions are reported [19,20], which suggests that the bacterium has colonized hosts with other geographic origin in some cases. Despite being genetically conserved, the JP2 genotype can be divided into different sequence types based on the presence of a number of point mutations in housekeeping genes [18]. One of these mutations, detected in *hbpA-2*, distinguishes microevolutionary subtypes of the JP2 genotype, found in individuals living in the Mediterranean area from those populations from West Africa [18].

Another *A. actinomycetemcomitans* genotype that lacks 640 bp in the leukotoxin promoter region has been isolated from a teenager of Ethiopian origin [21]. It is considered to be ‘JP2 genotype-like’, since it shares the 530 bp deletion with the JP2 genotype, but lacks additional 110 bp [21]. Within serotype b, there are other genotypes reported than those with the 530 and the 640 bp deletion, respectively [12,22]. Recently, a subgroup of highly virulent serotype b that consists of both JP2 and non-JP2 genotypes was identified [23]. A common feature for this genotype is high leukotoxicity, an identical arbitrarly primed (AP) polymerase chain reaction (PCR) pattern, and the presence of an intact *cagE* gene.

For the elucidation of the association of specific virulent bacterial species to periodontal disease, ‘the presence’ in the site of infection is a weak parameter. To strengthen this association, the so-called ecological plaque hypothesis should be followed [24]. This means that the proportion of specific pathogenic bacterial species at diseased sites has to be quantified.

Aiming to investigate the role of *A. actinomycetemcomitans* and its different virulent genotypes in periodontal disease, this study used the local collection of clinical isolates. In the Clinical Laboratory of the Dental School in Umeå, Sweden, various periodontitis-associated bacterial species have been isolated from clinical samples and have been studied for >30 years. The present study shows the colonization pattern of *A. actinomycetemcomitans* in two age groups of periodontitis patients. In addition, it focuses on the genetic characterization of isolates of *A. actinomycetemcomitans* and hypothesizes that the genetic diversity of the bacterium is of major importance for the colonization pattern among groups of younger and older periodontitis patients.

## Material and methods

### Study population and collection

The study collection comprises data from microbiological analyzes of 3,459 subgingival plaque samples collected from 1,445 patients, 337 ‘younger’ patients (YP; ≤35 years of age) and 1,108 ‘older’ patients (OP; >35 years of age) during 15 years (2000–2014). The clinical diagnosis of the patients was not homogeneously reported in the patient information attached to the referral to the laboratory for microbiological diagnostics. Thus, the classification of the patients was digitomized only and was based on the old definition of early onset periodontitis, which distinguished patients ≤35 years versus those >35 years of age [25]. Samples were sent from the student’s clinic and from the Specialist Clinic of Periodontology at the Dental School in Umeå, as well as from external specialist dental clinics throughout Sweden. The students collected samples from periodontal pockets >5 mm, measured from the top of the gingival margin to the bottom of the periodontal pocket. Samples from the specialist clinics of periodontology were collected from individuals suffering from periodontitis, although clinical and other parameters were sometimes unsystematically reported due to the many different clinics referring to the laboratories and to the retrospective nature of this study. The patients were between 9 and 92 years old and were sampled one to six times within the study period, and one to eight samples were taken each time. The ancestry of the patients, infected by the JP2 genotype of *A. actinomycetemcomitans*, was reported by the clinicians, except for two patients (patient 3 and 4; Table 1) who were ancestry tested [21].

**Table 1.** Proportion of the JP2 genotype of *Aggregatibacter actinomycetemcomitans* in samples collected from periodontis patients and characteristics of the carriers of this genotype

Isolate		Aa1	Origin	hbpA-22	M/W3	Age(years)
1	520A-01	57-90	Sweden	A	W	30
2	246A-04	0.1-25	Algeria	G	M	43
3	133A-08	78	Sweden	A	W	33
4	BL1-08	4	Sweden	G	M	62
5	090A-10	0.9-90	Sweden	G	M	27
6	196A-10	3.2-4.1	Croatia	G	M	23
7	115A-11	73-97	Iraq	G	M	16
8	352B-11	92	Iraq	G	M	23
9	245A-12	90	Sweden	G	M	18
10	557A-12	0.1-62	Cape Verde	A	W	19
11	338A-13	28-39	Sweden	G	M	31
12	342A-13	20	Morocco	G	M	15
13	408A-13	0.6-0.7	Gambia	A	W	15
14	456A-13^5^	42-68	Ethiopia	G	M	16
15	304A-14	0.3-2.7	Sweden	G	M	15
16	361A-14	76-98	Morocco	G	M	23
17	698A-14^5^	2.5-18	Morocco	G	M	16

% of total viable counts.

G and A are nucleotides within the hemoglobin-binding gene (*hbpA-2*) at position 525285 of the genome (HK1651) [18].

Mediterranean (M) or West African (W) origin of the JP2 genotype carriers

The proportion of *A. actinomycetemcomitans* could not be calculated due to contamination of the sample [20].

This isolate of *A. actinomycetemcomitans* has a 640 bp deletion within the leukotoxin promoter region [21].

Isolates of *A. actinomycetemcomitans* were collected from the patients and stored in a freezer. However, *A. actinomycetemcomitans* was not isolated or could not be recovered from the freezer from all positive patients, especially those examined during the first years of the study period. Thus, the study collection comprises isolates from 347 (78%) of the 435 *A. actinomycetemcomitans*–positive patients. Since 88 isolates are missing, the 347 isolates belong to a subpopulation of 1,357 patients.

### Quantification of *A. actinomycetemcomitans*

The samples were transported in an anaerobic medium (VMGAIII; Viable Medium of the Department of Bacteriology, University of Gothenburg) [26] to the Clinical Laboratory at the Dental School in Umeå, Sweden, serially diluted in a salt buffer [27], and spread on blood agar plates for the determination of the total number of bacteria. The dilutions were spread on Trypticase-Bacitracin plates for the determination of *A. actinomycetemcomitans* [28]. *A. actinomycetemcomitans* was identified according to established methods [28]. If the patients were sampled more than once, the sample containing the highest proportion of *A. actinomycetemcomitans* of the total viable count (TVC) were consequently selected for subsequent calculations.

### Characterization of *A. actinomycetemcomitans* isolates

All isolates were serotyped. In addition, all serotype b isolates were leukotoxin promoter and AP-PCR genotyped. For the preparation of the PCR mixtures, PureTaq Ready-To-Go PCR (GE Healthcare; Little Chalfont, UK) was used. For serotyping and leukotoxin promoter typing, DNA was isolated by heating suspensions of the isolates in water for 8 min, while for the AP-PCR, the DNA was purified with GenElute Bacterial DNA kit (Sigma–Aldrich, St. Louis, MO).

The primers used and the PCR program for the serotyping and leukotoxin promoter typing are described elsewhere [29–31]. For the AP-PCR genotyping, the random sequence oligonucleotide OPB-3 (AGTCAGCCAC; Invitrogen, Carlsbad, CA; 0.4 µM) was used. The concentration of MgCl_2_ in the PCR mixture was increased to 2.5 µM. The amplification was carried out as previously described [32].

The sequencing of the house-keeping gene, *hbpA-2*, was carried out as described elsewhere [18].

### Statistical analyses

Chi-square tests were used to identify significant differences in the distribution of the individuals and samples positive for *A. actinomycetemcomitans* in relation to the age group. The same test was used to examine differences in the distribution of *A. actinomycetemcomitans* AP-PCR types among YP and OP. The differences in the proportions of the bacterium in patients from the two age groups were calculated with the Mann–Whitney test. The rank test was used to calculate significant differences in the distribution of *A. actinomycetemcomitans* serotypes in samples from the two age groups.

## Results

### Presence of *A. actinomycetemcomitans*

*A. actinomycetemcomitans* could be isolated from around 30% of the sampled patients (Table 2). The detection frequencies of the bacterium were similar independently if the calculation was based on (a) the total number of subgingival plaque samples, or (b) the total number of patients (Table 2). Both ways for presentation showed that the detection frequency of the bacterium was significantly higher among YP than among OP (A; B: *p* < 0.001).

**Table 2.** Detection frequencies of *A. actinomycetemcomitans* (*Aa*) in subgingival plaque samples collected from younger (YP; ≤35 years) and older patients (OP; >35 years) with periodontitis

	A		B	
Age groups	***n***	***Aa***, ***n*(%)**	***n***	***Aa***, ***n*(%)**
YP	934	379 (40.6)	337	162 (48.1)
OP	2,535	529 (20.9)	1,108	273 (24.6)
YP + OP	3,459	908 (26.3)	1,445	435 (30.1)

*Aa* was significantly more prevalent among YP than among OP (A, B; *p *< 0.001). A, distribution of *Aa*-positive samples; B, distribution of *Aa*-positive patients.

### Proportions of *A. actinomycetemcomitans*

When all patients carrying *A. actinomycetemcomitans* were distributed into different groups based on the proportion of the bacterium in the samples, approximately 14% of the patients were observed in the group of samples containing >50% of the bacterium (Table 3). However, when the patients were distributed in the two age groups, samples containing > 50% *A. actinomycetemcomitans* were five times more common among YP than among OP.

**Table 3.** Distribution of patients, *n* (%), in groups with regard to age (YP and OP) and proportions (%) of *A. actinomycetemcomitans* of total viable count (TVC) in the samples from the patients (*n* = 435)

Age groups	>0-1%	>1-5%	>5-25%	>25-50%	>50%	*n*
YP	38 (23.5)	30 (18.5)	31 (19.1)	17 (10.5)	46 (28.3)	162
OP	108 (39.6)	51 (18.7)	79 (28.9)	20 (7.3)	15 (5.5)	273
YP + OP	146 (33.6)	81 (18.6)	110 (25.3)	37 (8.5)	61 (14.0)	435

Samples containing >50% *Aa* were significantly more common among YP than they were among OP (*p *< 0.001).

### Distribution of serotypes of *A. actinomycetemcomitans*

**Figure 1.**
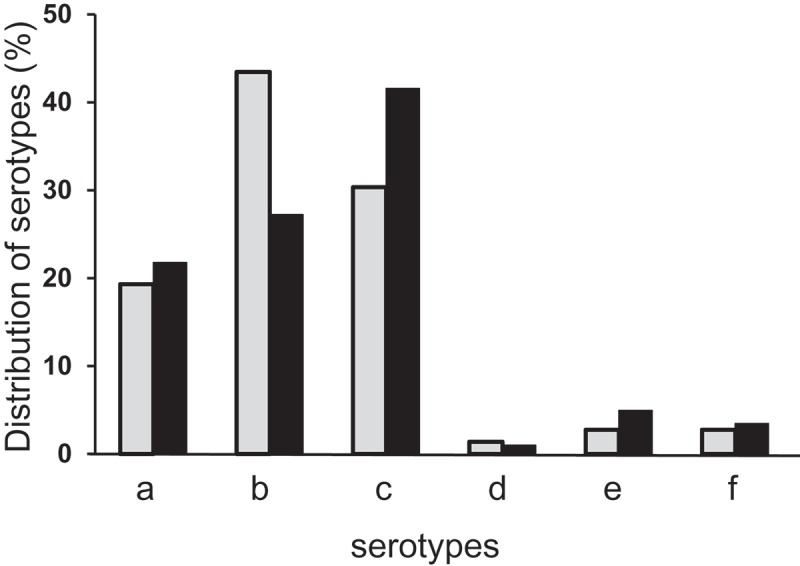
Percentage distribution of serotypes a–f of *A*ggregatibacter *actinomycetemcomitans* (*Aa*) among the isolates from younger patients (YP; gray columns; *n = *145) and older patients (OP; black columns; *n = *202). Serotyping of the collection of *Aa* isolates showed that serotype b was significantly more prevalent among YP, while serotype c was more prevalent among OP (*p* < 0.001).

Serotyping of the collection of *A. actinomycetemcomitans* isolates showed that serotype b was significantly more prevalent among YP, while serotype c was more prevalent among OP (*p* < 0.001). For the remaining serotypes, the distribution was similar in the two age groups (Figure 1).

When the three most prevalent serotypes of *A. actinomycetemcomitans* (a, b, and c) were distributed into proportions of the bacterium in the samples, serotype b was the most common in the category with the highest proportions (>50%) in both age groups. However, the prevalence of serotype b was almost five times higher among YP than in the corresponding category among OP (Table 4).

**Table 4.** Distribution of *A. actinomycetemcomitans-*isolates, *n* (%), in groups with regard to age (YP and OP), serotype (a, b, and c) and proportions (%) of *Aa* of TVC in the samples (*n = *317)

Age groups	>0–1%	>1–5%	>5–25%	>25–50%	>50%	*n*
YP						
a	7 (25.0)	3 (10.7)	7 (25.0)	2 (7.1)	9 (32.1)	28
b	8 (12.7)	5 (7.9)	14 (22.2)	6 (9.5)	30 (47.6)	63
c	18 (40.9)	11 (25.0)	8 (18.2)	4 (9.1)	3 (6.8)	44
OP						
a	11 (25.0)	10 (22.7)	14 (31.8)	6 (13.6)	3 (6.8)	44
b	18 (32.7)	9 (16.4)	20 (36.4)	2 (3.6)	6 (10.9)	55
c	35 (42.2)	20 (24.1)	22 (26.5)	5 (6.0)	1 (1.2)	83

### Prevalence and proportions of the JP2 genotype of *A. actinomycetemcomitans*

Of 1,357 patients, 17 (1.2%) carried the JP2 genotype of *A. actinomycetemcomitans* (Table 1). The proportion (% of TVC) of the JP2 genotype in at least one sample per patients was >50% in 56% of these 17 patients. Based on information from clinicians, 10 (59%) of the JP2 genotype carriers were considered to be of non-African origin, although ancestry testing had not been performed for all. Only two (12%) of the 17 JP2 carriers belonged to the OP group (Table 1). Furthermore, 13/17 (76.5%) JP2 genotype carriers were colonized by the Mediterranean subtype of the JP2 genotype.

### Distribution of AP-PCR genotypes of *A. actinomycetemcomitans*

**Figure 2.**
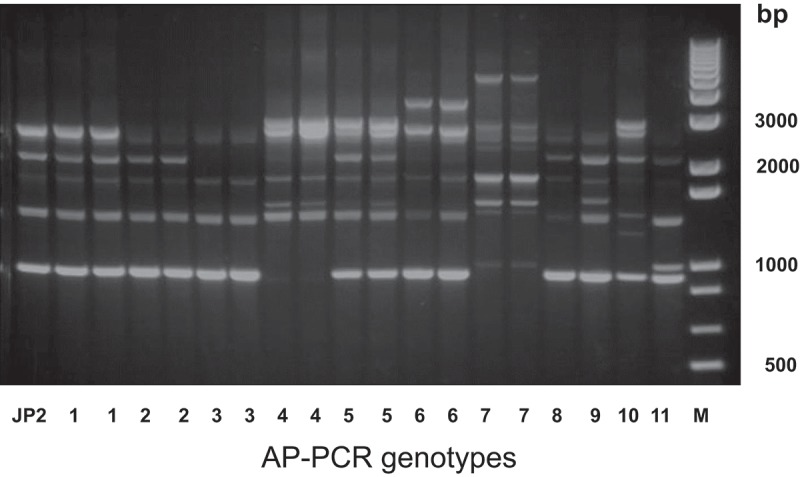
Different arbitrarily primed polymerase chain reaction (AP-PCR) genotypes of *A. actinomycetemcomitans* among serotype b isolates.

Eleven different AP-PCR patterns were identified among serotype b isolates (*n* = 119; Figure 2). Among those, 72 *A. actinomycetemcomitans* isolates belonged to AP-PCR genotypes 1 or 2 (Figure 2). The AP-PCR banding pattern of all 17 JP2 and JP2-like genotype isolates was identical (not shown), as compared to the JP2-reference strain HK1651, and belonged to AP-PCR genotype 1 (Figure 2). In Figure 2, AP-PCR genotypes 1–7 are each represented by two isolates, whereas for genotypes 8–11, only one isolate was available from each genotype.

**Figure 3.**
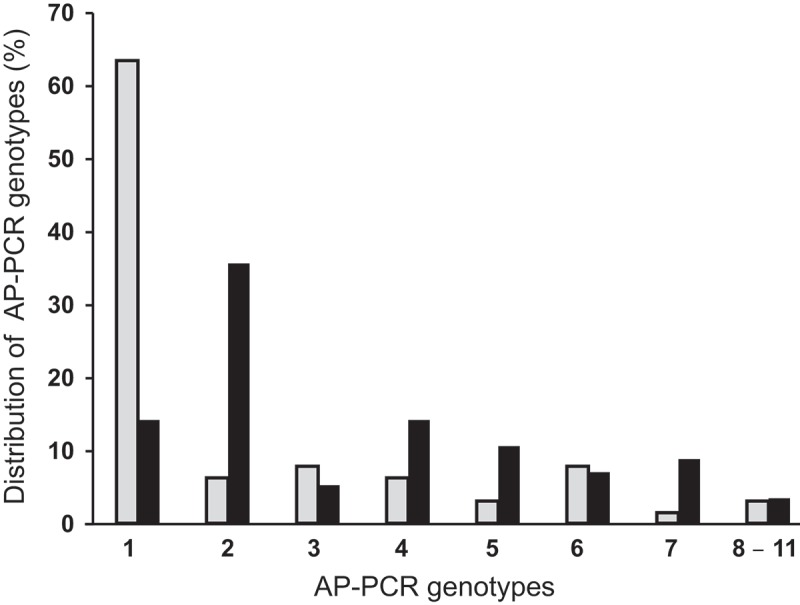
Proportions of serotype b isolates of *A. actinomycetemcomitans* from patients among YP (gray columns; *n = *63) and OP (black columns; *n = *56) when distributed in different AP-PCR genotypes, 1–11. AP-PCR genotype 1 was significantly more prevalent among YP than it was among OP (*p *< 0.001).

When the serotype b isolates were distributed among the AP-PCR genotypes with regard to the age of the carriers, >60% of the isolates from the YP group were identified as AP-PCR genotype 1, while only 15% of the isolates from the OP group were identified as AP-PCR genotype 1. In contrast, >35% of the isolates from the OP group were identified as AP-PCR genotype 2, while only 5% of the isolates from the YP group were identified as AP-PCR genotype 2 (Figure 3). In addition, independent of age, >50% of the AP-PCR genotype 1 isolates were distributed within the high proportion group, while none of the other AP-PCR type isolates was detected in this group.

## Discussion

A high number of reports on the prevalence and proportions of periodontitis-associated bacterial species in infected periodontal pockets are available [8,33,34]. However, the diversity of methods used in these and other studies complicates the comparison of results and conclusions [35–37]. Being aware of these problems, the present study shows, based on cultivation, that the detection frequency of *A. actinomycetemcomitans* was two times higher among YP than among OP living in Sweden. Furthermore, the characteristics of the *A. actinomycetemcomitans* isolates differed by the age of the patients.

In line with the ecological plaque hypothesis, this study also investigated the proportions of of *A. actinomycetemcomitans* in samples from periodontal pockets [24]. It is suggested that the capacity of *A. actinomycetemcomitans* to multiply more efficiently in periodontal pockets among YP could be due to enviromental factors. In deep pockets, which is one characteristic for periodontitis among OP, *A*. a*ctinomycetemcomitans* may be outcompeted by strictly anaerobic bacterial species such as *Fusobacterium nucleatum*, *Prevotella intermedia*, *Treponema denticola*, *Porphyromonas gingivalis*, and so on [38–41].

Six serotypes of *A. actinomycetemcomitans* (a–f) were detected among both YP and OP. However, serotype b, which is more virulent than the other serotypes [10,12], was more prevalent among YP. Serotype b seems more likely to survive in both younger and older patients in an environment that otherwise does not promote the existence of this bacterium. In other studies, the diversity among different serotypes in the binding to other bacterial species and to host molecules has been studied [42].

In the present study, 17 JP2 genotype carriers (i.e. 1.2% out of 1,357 patients) were detected. The majority of the JP2 genotype carriers were Caucasians, which is not in line with earlier reports that JP2 genotype carriers almost exclusively are found in individuals of African origin [18]. Almost 90% of the JP2 genotype carriers belonged to the YP group. Also, in other studies, the detection rate of the JP2 genotype among OP is low [19].

Spreading of the JP2 genotype between family members is reported [18,20]. Also, in this study, a family-related prevalence was detected. Three isolates (3, 4, and 5) were collected from a family comprising a mother and her two daughters, while two isolates (7 and 8) were collected from two brothers. The leukotoxin promoter region of one isolate (14) is missing 640 bp instead of 530 bp [21]. Taken together, it is surprising that a relatively high number of JP2 genotype carriers were identified among Swedish periodontitis patients. It is tempting to speculate that the carriage of the JP2 genotype is when young patients with bone loss visit the dentist. This suggests that it might be valuable to characterize *A. actinomycetemcomitans* at the subspecies level in isolates from these patients. Risk for a family-related spreading of the JP2 genotype should also be taken in consideration. Apparently, more studies are needed to identify carriers of the JP2 genotype in non-African populations, since it seems they have been underestimated.

When this study monitored the genetic diversity within serotype b isolates of *A. actinomycetemcomitans* with the AP-PCR technique, it was found that the majority of the isolates from the YP were distributed in the AP-PCR 1 genotype group, while the prevalence of isolates from OP were four times lower in this AP-PCR group. Since AP PCR genotype 1 is reported to be more leukotoxic than other AP-PCR genotypes, it is suggested that serotype b isolated from YP are more virulent than those that colonize OP [12]. This hypothesis is further supported by the fact that the JP2 genotype belongs to AP-PCR type 1. In addition, non-JP2 genotype isolates with an AP-PCR pattern that was identical with the AP-PCR pattern of the JP2 genotype were more prevalent among patients with localized aggressive periodontitis than among adult periodontitis patients [43].

Since younger individuals are at an enhanced risk of developing the aggressive form of periodontitis, it could be speculated that early colonization with *A. actinomycetemcomitans* of AP-PCR genotype 1 is a risk factor for developing the disease later in life. Further investigation of this genotype showed that the presence of an intact *cagE* gene identified as a marker for AP-PCR genotype 1 and was suggested as a target for early identification of risk individuals [23].

For diagnosis and treatment purposes, proportions of periodontitis-associated bacterial species in samples from periodontitis patients have been determined for many years [44–48]. Today, the utility of these analyzes is controversial [48]. However, in non-responsive cases, sampling and quantification of periodontitis-associated bacterial species such as *A. actinomycetemcomitans* may be helpful for the determination of more successful treatment strategies [49–51].

The results of the present study indicate that analysis of subgingival plaque samples from younger individuals with periodontal AL could be beneficial. Due to increased globalization and migration, a higher prevalence of young individuals with periodontal disease may occur in countries with earlier reported low prevalence of aggressive periodontitis.

In summary, the prevalence of *A. actinomycetemcomitans* was significally higher among YP than among OP. Furthermore, additional individuals of apparently non-African origin, colonized with the JP2 genotype of *A. actinomycetem-comitans*, have been identified in the present study. In addition, it was shown that a cluster of *A. actinomycetemcomitans*, with shared genetic characteristics, contained both the JP2 and the non-JP2 genotype. This cluster of the bacterium was significantly more prevalent among YP with periodontitis.
